# Heart Rate Complexity and Autonomic Modulation Are Associated with Psychological Response Inhibition in Healthy Subjects

**DOI:** 10.3390/e25010152

**Published:** 2023-01-12

**Authors:** Francesco Riganello, Martina Vatrano, Paolo Tonin, Antonio Cerasa, Maria Daniela Cortese

**Affiliations:** 1 S. Anna Institute, Via Siris 11, 88900 Crotone, Italy; 2 Institute for Biomedical Research and Innovation (IRIB), National Research Council of Italy (CNR), 98100 Messina, Italy; 3 Pharmacotechnology Documentation and Transfer Unit, Preclinical and Translational Pharmacology, Department of Pharmacy, Health Science and Nutrition, University of Calabria, 87036 Rende, Italy

**Keywords:** heart rate variability, inhibitory control, complexity index, entropy

## Abstract

Background: the ability to suppress/regulate impulsive reactions has been identified as common factor underlying the performance in all executive function tasks. We analyzed the HRV signals (power of high (HF) and low (LF) frequency, Sample Entropy (SampEn), and Complexity Index (CI)) during the execution of cognitive tests to assess flexibility, inhibition abilities, and rule learning. Methods: we enrolled thirty-six healthy subjects, recording five minutes of resting state and two tasks of increasing complexity based on 220 visual stimuli with 12 × 12 cm red and white squares on a black background. Results: at baseline, CI was negatively correlated with age, and LF was negatively correlated with SampEn. In Task 1, the CI and LF/HF were negatively correlated with errors. In Task 2, the reaction time positively correlated with the CI and the LF/HF ratio errors. Using a binary logistic regression model, age, CI, and LF/HF ratio classified performance groups with a sensitivity and specificity of 73 and 71%, respectively. Conclusions: this study performed an important initial exploration in defining the complex relationship between CI, sympathovagal balance, and age in regulating impulsive reactions during cognitive tests. Our approach could be applied in assessing cognitive decline, providing additional information on the brain-heart interaction.

## 1. Introduction

Heart rate variability (HRV) is a well-known index of autonomic control of the heart, but it is also linked to cognitive and emotional control [1,2] as well as autonomic dysfunctions that may precede the cognitive impairment [3]. The heart rate is directly controlled by the brain through the sympathetic and parasympathetic branches of the autonomic nervous system (ANS), defining the well-known central nervous system-ANS axis, also known as central autonomic network (CAN) [4,5,6,7]. The HRV then represents the output of the complex brain-heart interaction [5,8,9]. Inhibition, also known as inhibitory control, is the ability to suppress or regulate impulsive (or automatic) reactions, producing responses via attention and reasoning. This mental ability is part of executive functions and aids in goal-setting and anticipatory planning. Inhibitory control stops inappropriate behaviors and spontaneous reactions and replaces them with a more appropriate, well-thought-out response.

Response inhibition has been identified as common factor underlying the performance in all executive function tasks [10]. They are generally divided into inhibition and interference control, working memory, and cognitive flexibility [11,12]. The last is also called set-shifting, mental flexibility, or mental set-shifting.

Alerting, orienting, and executive monitoring of actions involve salient autonomic responses observable in pupillary reactivity [13], skin conductance [14], and heart rate variability [15,16]. Response inhibition is one of the main cognitive tasks that require the ability to suppress a pre-potent motor response by adjusting it rapidly as a function of environmental changes.

The high-frequency band of the HRV spectrum correlates to parasympathetic heart activity, which is crucial for the individual to have an efficient adaptability to changing environmental demands. The vagal response is also correlated to respiration. Changes in breathing patterns can impact heart rate (HR) and HRV, with a general decrease in respiratory frequency associated with an increase in heart period (i.e., a decrease in heart rate). The respiratory characteristic linked to HR is known as respiratory sinus arrhythmia (RSA) [17,18]. A decrease in vagal control (i.e., reduced HF-HRV) may suggest a lack of flexibility in responding to changing demands, limiting the spectrum of possible responses and hence restricting the person’s capacity to create appropriate responses and inhibit incorrect ones [5].

Forte and colleagues, in their review [3], refer that the most-reported HRV analysis during cognitive tests regards the high frequency (HF), low frequency (LF), and its LF/HF ratio. Moreover, studies showed that higher values in HRV entropy were related to cognitive performances [19,20].

Since the heart rhythm is not regular, the entropy analysis better represents its complexity and unpredictable variability [21,22]. Higher and lower entropy rates correlate to a more complex heartbeat sequence and a more regular and predictable heartbeat, respectively. Given the complexity of the brain-heart two-way interaction described by the CAN [7], the HRV entropy may be used to assess the system’s health. In fact, a more complex heartbeat sequence was found to indicate better reactivity to the external/internal stimulus [19,23,24,25].

In the frame of the HRV entropy analysis, the Multiscale Entropy (MSE) [26] was developed to investigate the information content in non-linear signals at different temporal scales (coarse-graining), generally using the Sample Entropy [27]. The Complexity Index (CI), a scalar score that permits gaining insights into the integrated complexity of the measuring system, is the sum of the entropies calculated for several coarse-graining scales. [26]. It was found that the heart rate complexity correlates with brain activity [19,28,29], and that the complexity in heartbeat dynamics grows with it and vanishes with stress.

This work aims to define, for the first time, the overall characteristics of HRV signals in the frequency and non-linear domains during the execution of a Go/NoGo task. This task is used to assess someone’s ability to regulate an inappropriate response among interfering visual stop signal (NoGo) by pressing a button whenever a Go signal stimuli is made [30]. The presented test is based on an adaptation of the rule shift card test. This well-known cognitive task assesses flexibility, inhibition abilities, and rule learning [31,32]. We want to assess how the response inhibition in the rule shift card test [31,32] is linked to a higher brain-heart interaction. In particular, we sought to determine the following: (1) if there are significant differences within and between baseline and task phases, in terms of entropy and spectral parameters, (2) the correlations between the errors in the two Go/noGo tasks and physiological parameters, and (3) the accuracy to predict the poor or good performance of the subjects using recorded HRV parameters in the baseline.

## 2. Materials and Methods

### 2.1. Subjects

Participants were recruited from Institute S.Anna, Crotone, Italy. The chosen participants had never used any drugs before. Before the experiment, all participants were instructed to refrain from smoking and consuming caffeine for four to six hours. The following criteria were specifically required for inclusion: (1) no evidence of dementia or depression symptoms according to DSM-V criteria; (2) no use of antidepressant, anxiolytic, or antipsychotic drugs that could affect cerebral blood flow; (3) right-handedness; and (4) absence of chronic medical conditions (heart disease, hypertension, or diabetes). According to these criteria, 36 healthy graduate subjects were enrolled in this study with median age 41 and interquartile range 16 (21 females age 36 ± 11 and 15 males age 43 ± 9). No significant differences were between males and females for age (Mann-Whitney test: Z = −1.912, *p* = 0.056). All participants had normal or corrected to normal vision and normal color vision. All the participants gave written informed consent. In accordance with the Helsinki Declaration, the study was approved by the Ethical Committee of Regione Calabria (n.ro 172 17 July 2020).

### 2.2. Procedure

The experiment was conducted in a dimly lighted, soundproof room. Participants completed a modified version of the traditional Go/NoGo activity based on the Rule Shift Cards [31,32,33], designed using Biotrace+, while seated in a comfortable chair (https://www.mindmedia.com/Herten/Nederland/ accessed on 5 November 2022).

In the last task, the subject must modify the strategy learned in the first task, memorizing the element that previously appeared and inhibiting the response when the square color is different from that of the previous square.

**Figure 1:**
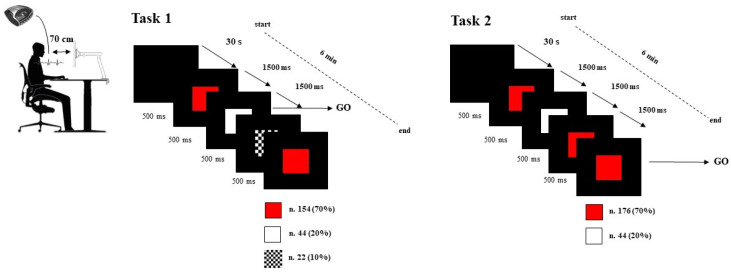
*Experimental detail*: Subject comfortably sits in front of the screen at 70 cm of distance from the monitor with the hand positioned near the spacebar of the computer keyboard. In the first task, the GO is represented by the white square, and the subject must hit the spacebar when it appears, and inhibit the response when the red square appears (the chess pattern with squares was the distractor). In the second task, the GO is represented by the appearance of a square with color equal to the previous square, while the NoGo is represented by a change in the appearance of the square color. The first square of the tasks appeared after 30 s of a black image. The stimulus duration was 500 ms and the interval of time between the stimuli was 1500 ms.

The protocol study consisted of a baseline (resting state condition) lasting 5 min and two different tasks. The tasks consisted of 220 visual stimuli with 12 × 12 cm red, white, and chess pattern squares on a black background. The first task included three distinct visual stimuli: red squares (frequent stimulus n.154—70%), white squares (rare stimulus n.44—20%), and a chess pattern with squares (distractor n.22—10%). It was based on fundamental response inhibition. A working memory response inhibition task made up the second. There were just red (common stimulus n.176—80%) and white (rare stimulus n.44—20%) squares in this instance. Each stimulus had a 1500 ms gap in between them, and each stimulus lasted 500 ms. The subject had to press the spacebar on the keyboard during the first task when the white square appeared, and during the second task when two successive visual stimuli of the same color were shown (Figure 1).

The subject was comfortably seated at a distance of 70 cm from the 24-inch monitor where the sequence of stimuli was displayed. There was no transient noise, and the environment was always the same temperature and brightness. The participant received assignment instructions prior to each activity.

### 2.3. Data Acquisition

The ECG recording was performed by NEXUS-32, and the stimuli were presented by Biotrace+ software (https://www.mindmedia.com/Herten/Nederland accessed on 5 November 2022). Since the ECG sample frequency rate of the signal acquisition can affect the HRV analysis, and a minimum sample of 250 Hz or higher are suggested [34,35], the signal was acquired at 256 Hz, and the 4 Hz cubic spline interpolation was applied for a correct extraction of the R-peaks.

### 2.4. Data Analysis

**Figure 2:**
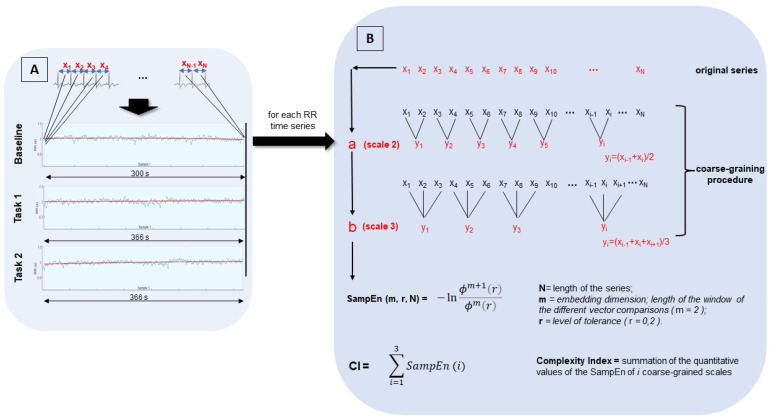
*Multiscale Entropy and Complexity Index Scheme.* (**A**) For each subject the tachogram was extracted in baseline, task 1, and task 2. N represents the length of each original detrended (trend line in red) time series (baseline N = 422 ± 100; task 1 N = 509 ± 83; task 2 N = 524 ± 102). (**B**) The sample entropy (SampEn) was calculated for the original and coarse-grained series A and B, setting the parameters m and r at 2 and 0.2, respectively. The complexity index (CI) was calculated as the sum of the SampEn of the scales 1 (original series), 2 (a), and 3 (b).

ECG was analyzed by Kubios advanced software for HRV analysis (v 3.1/Kuopio, Finland). The interpolation approach was used to eliminate artifacts and ectopic beats from the data. The CI (i.e., summation of the MSE from 1 to 3) and the natural logarithm of the HRV power of HF (0.15–0.5 Hz) (LnHF), LF (0.04–0.15 Hz) (LnLF), and LF/HF ratio were calculated. Heartbeat, like most physiological signals, is non-stationary due to the complex nature of the biological systems. It frequently contains either slow trends or very slow frequency oscillations. Since the HRV parameters may be affected by the non-stationarity of the signal, a quadratic polynomial model detrended the RR series to reduce the influence of lower frequencies in the power spectral density (PSD) results. The HF and LF PSD were calculated using the Fast Fourier Transform method (Welch’s PSD; windows width: 150 s). The transformation of the spectral power in their natural logarithm was applied because the measures showed a skewed distribution (i.e., skewness 2.48 ± 0.39; kurtosis 7.07 ± 0.77). The CI was based on the multiscale entropy (MSE) approach quantifying the degree of irregularity over a range of coarse-grained scales (τ) from 1 to 3. The interval between two consecutive R peaks of the QRS ECG complex represents the data points in the entropy analysis (Figure 2). The original series represents the scale for τ = 1. The coarse-grained scales 2 and 3 were constructed by averaging the IBI/tachogram’s data points within non-overlapping windows of increasing length τ (Figure 2). The sample entropy (SampEn) was calculated for each coarse-grained scale, and the CI was extracted by summing the sample entropy for each coarse-grained scale. Given a sample of length N, the SampEn is defined as the negative natural logarithm of the probability that if two sets of simultaneous data points of length m have distance < r, then two sets of simultaneous data points of length m + 1 also have distance < r [27]. Considering the length of our samples (i.e., original series: baseline N = 423 ± 101, task 1 N = 509 ± 83, task 2 N = 525 ± 102), the parameters m (i.e., embedding dimension; length of the window of the different vector comparisons) and r (i.e., level of tolerance: generally ranging from 0.1 to 0.25, corresponding to the 10–25% of the standard deviation of the series of data onto analysis) of SampEn were set to 2 and 0.2, respectively [27,36], for the original and the coarse-grained scale 2 and 3 (i.e., r was the 20% of the standard deviation of the original and rescaled time series).

### 2.5. Statistical Analysis

The non-parametric exact test was used for the statistical analysis. This approach provides more accurate results when the sample size is small or in the case of tables sparse or imbalanced. By the Wilcoxon exact test, the HRV parameters (LnLF, LnHF, LF/HF, SampEn for the original and the CI were compared in the different tasks. The effect size r was calculated as the absolute value of Z√(2N) (Wilcoxon’s test), where Z is the Z statistic of the statistical test and N is the total number of subjects. The effect size results were considered as follows: r < 0.1 not significant; 0.1 ≤ r < 0.3 low; 0.3 ≤ r < 0.5 medium; r ≥ 0.5 high. The level of significance was set at *p* ≤ 0.05.

The Pearson correlation test analyzed the correlation between HRV parameters and performance levels (errors and Reaction Time).

Because of outliers, the median of total errors was used to divide the subjects into Good Performance (GP) and Poor Performance (PP) groups. Good or poor performance was predicted by binary logistic regression. The backward approach in the logistic regression was used to select the regressors in the model, inserting the performance (i.e., GP and PP classification) as dichotomic variable, and age and HRV parameters (i.e., C.I., LnLF, LnHF, and LF/HF) as independent variables. The backward approach uses the Wald tests’ results for the regressor’s elimination, removing the variable with the least significant effect that does not meet the level for staying in the model [37]. The removed effect is excluded from the model, and the process is repeated until no other effect in the model meets the specified level for removal. The model’s accuracy was also checked, controlling the variables for collinearity by tolerance and its reciprocal variance inflation factor (VIF) [38].

## 3. Results

### Behavioral Data

The inhibitory task was successfully completed by all subjects. Table 1 reports the response time (RT) for GO trials and the percentage of errors for GO and NOGO trials. The RT and numbers of errors in Go and NoGo conditions raised in Task 2, as aspected.

**Figure 3:**
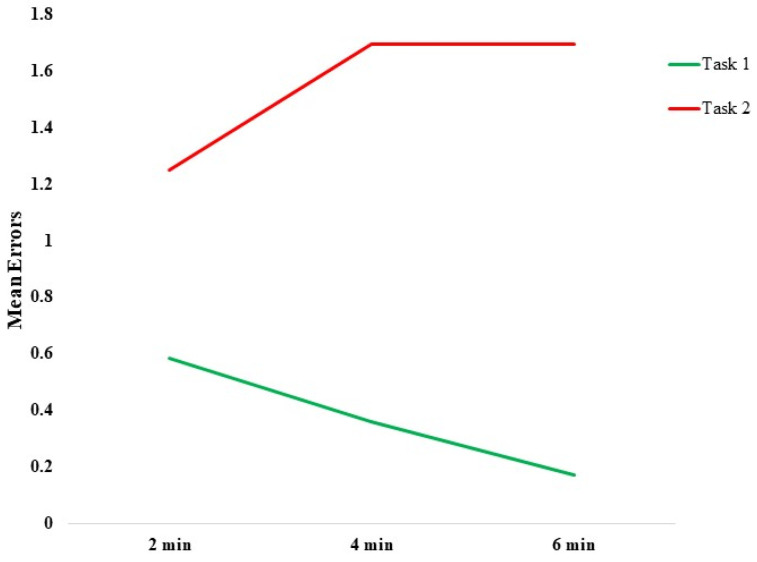
Mean errors during the tasks: number of errors during task 1 (green line) and task 2 (red line) along the time.

The number of errors were lower in task 1, with a decrease over time. Conversely, they increased over time in task 2 (Figure 3).

**Figure 4:**
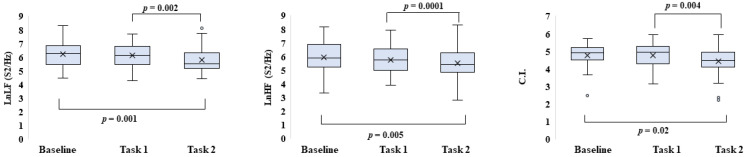
*Boxplot of the HRV parameters*: Boxplot of the natural logarithm of low-frequency power (LnLF) and high-frequency power (LnHF), and Complexity Index (CI) in resting-state (baseline) and during task 1 and task 2.

In the group, significant differences between baseline and Task 2, with a decreasing trend, were found for LnLF (Z = −3.189, *p* = 0.001, r = 0.38), LnHF (Z = −2.726, *p* = 0.005, r = 0.32) and CI (Z = −2.317, *p* = 0.02, r = 0.27). The same parameters showed significant differences comparing task 1 and task 2 (−3.473 ≤ Z ≤ −2.789, 0.0001 ≤ *p* ≤ 0.004, 0.33 ≤ r ≤ 0.41) (Figure 4).

**Figure 5:**
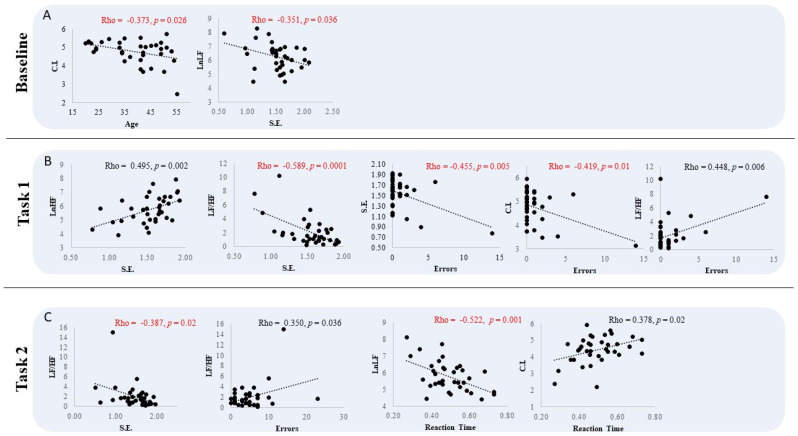
*Correlations among HRV parameters and performance:* Correlation among HRV parameters (SampEn (SE), Complexity Index (CI), HF power (LnHF), LF power (LnLF), LF/HF), performance levels (errors and Reaction Time) and age of the group. In red and black are the negative and positive correlations, respectively. (**A**) baseline; (**B**) task 1; (**C**) task 2.

In baseline, CI had a negative correlation with age (Rho = −0.373, *p* = 0.026) and the SampEn with the LnLF (Rho = −0.351, *p* = 0.036). In Task 1, the SampEn had a positive correlation with LnHF (Rho = 0.495, *p* = 0.002) and negative correlation with LF/HF (Rho = −0.589, *p* = 0.0001), while CI and SampEn had a negative correlation with the errors (Rho = −0.455, *p* = 0.005 and Rho = −0.419, *p* = 0.01, respectively) and positive correlation with LF/HF (Rho = 0.448, *p* = 0.006). In Task 2, LF/HF had a negative correlation with SampEn (Rho = −0.387, *p* = 0.02) and positive correlation with the errors (Rho = 0.350, *p* = 0.036), while the Reaction Time had a negative correlation with the LnLF (Rho = −0.522, *p* = 0.001) and positive correlation with the CI (Rho = 0.378, *p* = 0.02). Excluding the outlier from the analysis overall patterns of significant findings did not change (Figure 5).

Because of the presence of outliers by the median of the total errors (median of errors = 5) the subjects were divided in two sample groups: Good Performance (GP) (20 subjects; seven males aged 44 ± 7; 13 females age 35 ± 11; median of errors ≤ 5) and Poor Performance (PP) (16 subjects; eights males aged 43 ± 10; eight female age 39 ± 11; median of errors > 5).

The binary logistic regression was used to observe the probability of predicting the performances (i.e., GP/PP) from the HRV recorded values in the baseline. By backward stepwise binary logistic regression, age, CI, and LF/HF ratio were selected as regressors to classify GP and PP. No collinearity was among the selected variables (i.e., tolerance ≥ 0.96, VIF ≤ 1.8). The extracted model (GP/PP = −9,35 + 0.036 × age + 1.375 × CI + 0.597 × LF/HF; significance of the model (omnibus test) *p* = 0.032; Cox & Snell’s R2 = 0.22; Naghelkerte’s R2 = 0.29; Hosmer & Lemeshow’s test *p* = 0.32) classified correctly the GP (16/20; 80%) and PP (10/16; 63%) with a sensitivity of 73%, specificity of 71%, and balanced accuracy of 71%.

## 4. Discussion

We demonstrated that during response inhibition tasks, HRV metrics (LF, HF, SampEn, CI, and LF/HF ratio) are characterized by significant changes as a function of performance. In particular, we found that the following: (1) the CI and the logarithmic values of LF and HF decreased from baseline to task 2; (2) in baseline, the CI correlated negatively with age; (3) in task 2 the CI is positively correlated with the reaction time, and the LF/HF ratio is positively correlated with the errors; finally, (4) in baseline age, CI and LF/HF ratio are predictors of better performance (i.e., fewer numbers of errors) in the tests.

Several works report higher levels of HRV associated with better emotional responses [39], attentional [2,40], and executive performances [1,32,41,42], while lower HRV was associated with dysfunctional attitudes [43]. Studies found that in healthy subjects, a greater HRV in the resting state predicted better performance in tasks related to executive brain function [1,44,45], with similar results in psychopathological populations [1,45]. In resting-state conditions, less efficient task performance and lower response accuracy in pleasant and unpleasant Go trials were associated with lower HRV values [46]. Ottaviani and colleagues [32] demonstrated that higher parasympathetic levels predicted performance during the Rule Shift Cards and the Hayling Sentence Completion inhibitory tasks.

We found that LnLF and LnHF power and CI decreased from baseline to task 2. The results are consistent with the reactivity or the possible state of stress during mental fatigue [22,29,47,48,49].

Several studies reported the correlation between autonomic function and cognition, observing a higher parasympathetic modulation associated with better performance on tasks involving executive function [1,50,51,52]. However, knight and colleagues suggest that both sympathetic and parasympathetic should be considered together to observe changes in cognitive functions [53]. Indeed, a better cognitive performance was associated with increased sympathetic activity but not decreased vagal activity in older patients [54]. In our study, in baseline LnLF was negatively correlated with the entropy, and in task 1 and 2 the LF/HF ratio positively correlated with the errors (i.e., increases in the parasympathetic tone).

A decrease in the HRV complexity was correlated to major depressive disorders [20] and during cognitive tasks in anxious subjects [55]. Cardiac complexity was found to be particularly effective in differentiating between active, effortful emotion regulation and less effortful control and dysregulation [56]. In the field of disorders of consciousness, the CI was found to be a useful marker to discriminate unresponsive wakefulness syndrome/vegetative state patients from minimally conscious state patients [25], with higher CI correlated with a higher level of consciousness. Again, HRV complexity diminishes with age and disease [57,58], while MSE research in healthy persons over 40 revealed an age-related fall in heart rate complexity [59,60].

We found the CI negatively correlated with the errors in task 1 and positively correlated with the reaction time in task 2. Bakhchina and colleagues found that higher HRV entropy, measured by the Sample Entropy, is associated with a more complex response in goal-directed behavioral [61]. In particular, our results highlight that a higher CI is linked to less impulsivity and fewer errors, indicating a more rich complexity in the brain-heart interaction [62]. Moreover, we found that higher reaction time is associated with higher CI and lower LF. These results suggest that a higher brain-heart interaction is related to higher flexibility and adaptation at the rule shift. Moreover, we found that the CI is negatively correlated with age.

These findings supported the relationship between sympathovagal control, CI, and age, due to a reduction in the complexity of the physiologic dynamics associated with aging [63,64] and their involvement in the test performance. Moreover, the negative correlation between the SE with LF/HF ratio highlights the effect of the sympathetic response on the heart rate complexity. Porta and colleagues correlated the increase in sympathetic activity with decreased HRV entropy [65]. The sympathetic activity was found to increase during mental stress induced by various methods in healthy subjects [66,67]. The effect induced by the increased sympathetic activity is an increase in the heart rate and regularity of the heartbeats producing a decrease in the heart rate complexity [68,69].

Furthermore, CI, age, and LF/HF ratio were the regressors involved in the logistic model, which allowed in baseline to predict the performances with sensitivity and specificity of 73% and 72%, respectively.

To the best of our knowledge, in this study, a significant correlation between CI and inhibitory performance has been described for the first time. In particular, we found that the increased HRV entropy was linked to increased reaction time and decreased LF/HF ratio. The HRV complexity better identifies considerable changes in autonomic regulation providing new insight into changes under various physiological and pathological conditions, complementing the analysis in the time and frequency domain [70,71,72,73]. It has been demonstrated that the CI provided novel information on the brain-heart interaction in patients with disorders of consciousness [25,74,75]. Our data could provide new markers to assess the performances in cognitive tests in healthy and pathological subjects.

However, the relatively small sample size represents the limitations of this study. Moreover, the HRV decreases with the age [76,77], and as well as it was documented, the menstrual cycle can influence the HRV in women and also its non-linear property [78]. Again, a recording of 300 s for the baseline and 366 s for the tasks could represent a limit in calculating the multiscale entropy. However, the sample entropy has also been calculated in short data points (i.e., N = 100; [36]). A bigger sample size could better explain the outlier observed in Figure 2, where a male subject over 40 increased the LF/HF values from 5.5 in baseline to 14.9 in task 2. The outlier does not change the significance of the results. However, it is interesting to observe that the high error numbers are associated with increased HR from baseline to task 2 and that while LF did not change, the HF decreased. This highlights the importance of HF autonomic modulation during cognitive tests [79].

On the other hand, the strength of this study was the evaluation of the non-linear proprieties of the HRV during all phases (baseline, tasks 1 and 2). In this way, it has been possible to observe differences in the performance non-related to a significant difference in the vagal modulation of the ANS but to a different modulation in the brain-heart two-way interaction. Further studies should consider the ECG and EEG simultaneous recording to understand the brain-heart two-way interaction better. Moreover, the use of novel methods in the entropy analysis, such as Refined MSE, the Linear MSE, or the Modified MSE [80,81], could better explain this complex interaction. Moreover, the analysis of the respiratory sinus arrhythmia could provide further information about how vagal system mediates the cognitive performance.

Our study performed an important initial exploration in defining the complex relationship between CI, sympathovagal balance, and age in inhibitory function.

Our approach could be applied in assessing cognitive decline and as a complement to the EEG and fMRI analysis, providing additional information on the brain-heart interaction.

## Figures and Tables

**Table 1:** Behavioral Variables.

	Task 1	Task 2
	Mean ± SD	Mean ± SD
RT during GO trials (s)	0.37 ± 0.08	0.49 ± 0.11
% errors during GO trials	0.04%	1.38%
% errors during NoGo trials	1.81%	2.93%

RT: Reaction Time; SD: Standard Deviation.

## Data Availability

The data presented in this study are available on request from the corresponding author.
